# Prevalence of extended spectrum beta lactamase and molecular detection of blaTEM, blaSHV and blaCTX-M genotypes among Gram negative bacilli isolates from pediatric patient population in Gaza strip

**DOI:** 10.1186/s12879-023-08017-1

**Published:** 2023-02-20

**Authors:** Nabil Abdullah El Aila, Nahed Ali Al Laham, Basim Mohammed Ayesh

**Affiliations:** 1 Department of Medical Laboratory Sciences, Faculty of Medical Sciences, Al-Aqsa University, Gaza, Palestine; 2 Department of Laboratory Medicine, Faculty of Applied Medical Sciences, Al Azhar University, Gaza, Palestine; 3 Department of Medical Laboratory Sciences, Faculty of Applied Sciences, Al-Aqsa University, P.O. box. 4051, Gaza, Palestine

**Keywords:** Gram-negative bacilli, Extended-spectrum beta-lactamase, CTX-M, TEM, SHV, Antimicrobial susceptibility, Pediatric, Gaza strip

## Abstract

**Background:**

Extended-spectrum *β* lactamases (ESBLs), have the ability to hydrolyze and cause resistance to various types of the *β*-lactam antibiotics, including the extended-spectrum (or third-generation) cephalosporins (e.g., cefotaxime, ceftriaxone, ceftazidime) and monobactams (e.g., aztreonam). ESBL-producing Gram negative bacteria is still posing significant therapeutic challenges.

**Objectives:**

To assess the prevalence and molecular characteristics of ESBL producing Gram negative bacilli, isolated from a cohort of pediatric patients in Gaza hospitals.

**Methods:**

A total of 322 isolates of Gram-negative bacilli were collected from four referral pediatric hospitals in Gaza, namely: Al-Nasr, Al-Rantisi, Al-Durra and Beit Hanoun hospitals. These isolates were tested for ESBL production using the double disk synergy and CHROMagar phenotypic methods. Molecular characterization of the ESBL producing strains was performed using PCR targeting the CTX-M, TEM and SHV genes. Antibiotic profile was done using Kirby Bauer method according to Clinical and Laboratory Standard Institute.

**Results:**

Out of 322 isolates tested by phenotypic methods, 166 (51.6%) were ESBL positive. The prevalence of ESBL production in Al-Nasr, Al-Rantisi, Al-Durra and Beit Hanoun hospitals was 54%, 52.5%, 45.5% and 52.8% respectively. The prevalence of ESBL production among *Escherichia coli*, *Klebsiella pneum*oniae, *Pseudomonas aeruginosa*, *Acinetobacter* spp., *Proteus mirabilis*, *Enterobacter* spp., *Citrobacter* spp., and *Serratia marcescens* is 55.3%, 63.4%, 17.8%, 57.1%, 33.3%, 28.5%, 38.4%, and 4% respectively.

ESBL production among urine, pus, blood, CSF and sputum was 53.3%, 55.2%, 47.4%, 33.3%, and 25% respectively. Out of the 322 isolates, 144 were screened for CTX-M, TEM and SHV production. Using PCR, 85 (59%) had at least one gene. The prevalence rate of CTX-M, TEM and SHV genes was 60%, 57.6%, and 38.3% respectively.

Meropenem and amikacin were highest rates of susceptibility antibiotics against ESBLs producers (83.1% and 82.5% respectively), while the least effective antibiotics were amoxicillin (3.1%) and cephalexin (13.9%). Moreover, ESBLs producers showed high resistance rate to cefotaxime, ceftriaxone and ceftazidime (79.5%, 78.9% and 79.5% respectively).

**Conclusion:**

Our results show high prevalence of ESBL production among Gram negative bacilli isolated from children in different pediatric hospitals in Gaza strip. A substantial level of resistance to first and second generation cephalosporins was also observed. This ascertains the need for a rational antibiotic prescription and consumption policy.

## Background

Extended-spectrum *β*-lactamases (ESBLs) are a predominant cause of *β*-lactam resistance in Gram-negative bacilli (GNB) [1, 2]. The incidence and prevalence of infections caused by ESBLs producing GNB is increasing worldwide, both in the healthcare as well as community settings, thereby posing significant therapeutic challenges [3–5]. ESBLs are plasmid-mediated groups of enzymes that hydrolyze penicillins, extended-spectrum cephalosporins, and aztreonam [6, 7]. ESBLs are caused by production of SHV, TEM and CTX-M encoded by the *bla*_SHV_, *bla*_TEM_, and *bla*_CTX-M_ genes respectively. There have been almost 300 distinct ESBL variations described [8]. Despite the fact that TEM and SHV variants are the most common ESBLs, strains expressing CTX-M ESBLs have emerged in many countries over the last decade [9, 10] and are currently the most common non-TEM, non-SHV ESBL type.

Among Enterobacteriaceae, ESBLs have been found mostly in *Klebsiella* spp. and *Escherichia coli* as well as in other Enterobacteriaceae families such as *Enterobacter* spp., *Proteus* spp., *Citrobacter* spp., *Morganella* spp., *Providencia* spp., *Salmonella* spp., and *Serratia* spp. [3, 11, 12]. ESBL-producing Gram negative bacteria is causing greater use of other expensive antimicrobials (such as carbapenems), prolonged hospital stays, increasing morbidity, mortality and health care costs [13]. ESBL-producing bacteria may simply colonize the gastrointestinal tract of children [14, 15]. They are also associated with disease in both adults and children including infections of the urinary tract, abdomen, and bloodstream [16, 17]. Treatment options for multidrug resistance (MDR) Gram-negative bacterial infections are generally limited. Due to the fact that fewer antibiotics have been licensed for use in children, in addition to the perpetual dearth of pediatric drug trials, the problem must be addressed and followed up [18].

The prevalence of infections caused by ESBL-producing bacteria in children is increasing worldwide, including both developed and developing nations [17]. Of concern is the relatively high mortality rate that has been described in outbreaks due to ESBL-producing organisms. ESBL-producing bacteria may colonize the gastrointestinal tract of children [14, 15].

The emergence of ESBL producing organisms is a growing problem in general pediatric practice [19]. This limits the options of previously effective antibiotics, resulting in poorer outcomes [20]. Since the production of ESBL confers resistance to most cephalosporins, the choice of antibiotics used in infections caused by these organisms relies mostly upon carbapenems [21, 22].

The present study was undertaken to determine the prevalence of ESBL in pediatric hospitals in Gaza strip using phenotypic and molecular tools. To our knowledge, no data was previously published addressing the prevalence of ESBL among pediatric population in Gaza strip.

## Materials and methods

### Study design

A cross-sectional study involving 322 clinical isolates of Gram negative bacilli that were collected from different pediatric hospitals including Al-Nasr, Al-Rantisi, Al-Durra and Beit Hanoon hospitals in Gaza strips.

The sample sources of these isolates were urine, pus, sputum, blood and ear discharges.

These clinical isolates were collected from (February–May 2019). The specimens which has been included in this study was from pediatric and hospitalized patients. Outpatients and adult’s samples were not included in this study.

The study was approved by the department of human resources and development in the Ministry of Health—Gaza.

### Culture of clinical isolates

All collected clinical isolates were sub-cultured on MacConkey agar and incubated overnight at 37 °C aerobically. Bacterial isolates were subjected for identification upon cultural characteristics and other relevant biochemical reactions. Moreover, DNA extraction and antibiotic susceptibility testing were performed.

### Antimicrobial susceptibility testing

Antibiotic susceptibility testing was performed by modified Kirby Bauer disc diffusion method according to the Clinical and Laboratory Standards Institute (CLSI) 2018 [23] using Mueller–Hinton agar. Prior to inoculation, the swab stick was dipped into bacterial suspension having visually equivalent turbidity to 0.5 McFarland standards. The antibiotics used in this study were Amoxicillin–Clavulanic acid (20/10 µg), Cefotaxime (30 µg), Ceftriaxone (30 µg), Ceftazidime (30 µg), Gentamicin (10 µg), Amikacin (30 µg), Ciprofloxacin (5 µg), Imipenem (10 µg), Amoxicillin (30 µg), Cephalexin (30 µg), Cefuroxime (30 µg), Co-Trimoxazole (25 µg), Meropenem (10 µg) and Doxycycline (30 µg). Zone of inhibition for each antimicrobial agent was interpreted, reporting the organism as resistant, intermediate or susceptible.

### Phenotypic detection of ESBLs using double disk synergy test

Phenotypic confirmatory test for ESBL producers was done using the double disc synergy test. The organism to be tested was spread onto a Mueller–Hinton agar plate. The antibiotic Ceftriaxone (30 µg), Cefotaxime (30 µg), Ceftazidime (30 µg), and Amoxycillin/Clavulanic acid (20/10 µg) were placed at distances of 20 mm (edge to edge) from the Amoxycillin/Clavulanic acid disc that was placed in the middle of the plate. After 24-h incubation, if an enhanced zone of inhibition between any of the Cephalosporin antibiotics and the Amoxycillin/Clavulanic acid disc occurred, the test was considered positive. This indicated synergistic activity with Clavulanic acid and the presence of an ESBL. The quality assurance was performed weekly using *K. pneumoniae*, ATCC number 700603 (ESBL producing isolate) and *E. coli* ATCC number 25922 (susceptible isolate) as positive and negative controls, respectively [24].

### Phenotypic detection of ESBLs using CHROMagar ESBL

The CHROMagar ESBL provided by CHROMagar (Paris, France) was obtained from the supplier as a prepared plate medium. Each bacterial strain was cultured on CHROMagar ESBL, and was incubated aerobically at 37 °C for 18–24 h. Colonies of ESBL producers develop species-specific colors (dark pink to reddish coloration for *E. coli*; metallic blue coloration for *Klebsiella* spp.; and a brown halo for *P. mirabilis*). Non-ESBL producers grow with colorless colonies or not at all on CHROMagar ESBL [25].

### DNA extraction

DNA was extracted from cultured isolates by alkaline lysis as previously described [26]. Briefly, one bacterial colony was suspended in 20 µl of lysis buffer (0.25% sodium dodecyl sulfate, 0.05 N NaOH) and heated at 95 °C for 15 min. The cell lysate was diluted by 180 µl sterilized distilled water. The cell debris was pelted by centrifugation at 16,000×*g* for 5 min. and the supernatants were used for PCR or frozen at − 20 °C until further use.

### Detection of TEM, SHV and CTX-M genes by PCR

The sequences of primers used for detection of SHV gene were 5′-GCC CGG GTT ATT CTT ATT TGT CGC-3′ as a forward primer and 5′-TCT TTC CGA TGC CGC CGC CAG TCA-3′ as a reverse primer. The two primers produce a 1016 bp fragment [27]. For detection of CTX-M gene, the sequences of primers used were 5′-ACC GCG ATA TCG TTG GT-3′ as a forward primer and 5′-CGC TTT GCG ATG TGC AG-3′ as a reverse primer. The two primers produce a 550 bp fragment [28].

For detection of TEM gene, the sequences of primers used were 5′-ATG AGT ATT CAA CAT TTC CG-3′ as a forward primer and 5′-CCA ATG CTT AAT CAG TGA GG-3′ as a reverse primer. The two primers produce a 858 bp fragment [29].

The reactions were performed in 25 µl final volumes in the presence of 1µM of each primer, 2 µl DNA and 1× of the GoTaq® Green Master Mix (Promega, USA). The thermal cycling program for detection of SHV, CTX-M and TEM genes was as follows: one cycle of initial denaturation at 95 °C for 5 min; 34 cycles of denaturation at 95 °C for 30s, the proper annealing temperature (54 °C for SHV, 55 °C for CTX-M or 68 °C for TEM) for 30s, and extension at 72 °C for 1 min; followed by a final extension at 72 °C for 5 min. The amplified products were resolved on a 2% agarose gel. The fragments were stained with ethidium bromide and visualized and photographed using gel documentation system. A 100 bp ladder was run as a molecular weight marker (Bioline —UK) A water sample was run as a blank negative amplification control in each run to exclude contamination.

### Statistical analysis

The results were tabulated and analyzed using version 20 of the Statistical Package for the Social Sciences (SPSS). Frequencies, cross tabulation and the Chi-square test and fisher exact test were performed to determine statistical significance at *P-*value of less than 0.05.

## Results

### Phenotypic characterization

During the study period, a total of 322 isolates of Gram-negative bacilli were collected from the four pediatric hospitals in Gaza.

The number of isolates collected from Al-Nasser, Al-Rantisi, Al-Durrah and Beit Hanoun hospitals was as 150 (46.6%), 59 (18.3%), 77 (23.9%) and 36 (11.1%) respectively. Eight different Gram negative bacteria were isolated. including *E. coli*, *Klebsiella pneumoniae*, *Pseudomonas aeruginosa*, *Acinetobacter* spp., *P. mirabilis*, *Enterobacter* spp., *Citrobacter* spp., and *Serratia marcescen*. These isolates were identified depending on morphology, cultural characteristics and biochemical reactions. These isolates were characterized by their antibiogram, ESBL production using double disc synergy test and detection of the ESBL genes TEM, SHV, and CTX- M.

Out of the 322 isolates, 166 (51.6%) were positive for ESBL production whereas 156 (48.4%) were non-ESBL. The prevalence rate of ESBL production was 54% in Al-Nasser, 52.5% in Al-Rantisi, 45.5% in Al-Durrah and 52.8% in Beit Hanoun hospital.

The frequency of *E. coli*, *Klebsiella pneumoniae*, *Pseudomonas aeruginosa*, *Acinetobacter* spp., *P. mirabilis*, *Enterobacter* spp., Citrobacter spp., and *Serratia marcescens* was (52.7%, 20.4%, 8.6%, 6.5%, 2.7%, 4.3%, 4% and 0.3%) respectively (Table 1).

**Table 1:** Distribution of isolates by type of microorganism and prevalence of ESBL.

	Al-Nasser		Al-Rantisi		Al-Durrah		Beit Hanoun		Total	
	N	%	N	%	N	%	N	%	N	%
Frequency	150	46.6	59	18.3	77	24	36	11.1	322	100
ESBL	81	54	31	52.5	35	46	19	52.8	166	51.6
*E. coli*	74	49.3	34	57.6	43	56	19	52.8	170	52.7
*K. pneumoniae*	33	22	10	16.9	17	22	6	16.6	66	20.4
*P. aeruginosa*	14	9.3	8	13.5	3	3.8	3	8.3	28	8.6
*Acinetobacter* spp.	11	7	3	5	6	7	2	5.5	21	6.5
*P. mirabilis*	4	2	2	3	2	2.5	1	2.7	9	2.7
*Enterobacter* spp.	7	4.6	1	1.6	4	5.1	3	8.3	14	4.3
*Citrobacter* spp.	6	4	1	1.6	2	2.5	2	5.5	13	4
* S. marcescens*	1	0.6	0	0	0	0	0	0	1	0.3

As shown in Table 2, *K. pneumoniae* was the leading ESBL producing bacteria isolated from Al-Nasser, Al-Rantisi, Al-Durrah and Beit Hanoun hospital. The prevalence rate of ESBL production was 55.3% for *E. coli*, 63.6% for *K. pneumoniae*, 17.8% for *P. aeruginosa*, and 57.1% for *Acinetobacter* spp., 33.3% for *P. mirabilis*, 28.5% for *Enterobacter* spp., 38.4% for *Citrobacter* spp. and the only one *Serratia* isolate was ESBL producer (Table 2).

**Table 2:** Distribution of ESBls by type of becatrial isolate

	ESBL	Non ESBL	Total	% ESBL
*Escherichia coli*	94	76	170	55.30%
*Klebsiella pneumoniae*	42	24	66	63.60%
*Pseudomonas aeruginosa*	5	23	28	17.80%
*Acinetobacter* spp.	12	9	21	57.1
*Proteus mirabilis*	3	6	9	33.3
*Enterobacter* spp.	4	10	14	28.5
*Citrobacter* spp.	5	8	13	38.4
*Serratia marcescens*	1	0	1	100%
Total	166	156	322	

The distribution of isolates by type of specimen was 79.2% urine, 9% pus, 5.9% blood, 0.9% CSF and 5% sputum (Table 3). The prevalence of ESBL among these specimens was 53.3%, 55.2%, 47.4%, 33.3%, and 25% respectively.

**Table 3:** Distribution of isolates by type of specimen

	Al-Nasser		Al-Rantisi		Al-Durrah		Beit Hanoun		Total	
	N	%	N	%	N	%	N	%	N	%
Urine	112	74.6	42	71.2	72	93.4	29	80.5	255	79.2
Pus	14	9.4	6	10.2	4	5.4	5	13.9	29	9
Blood	18	12	1	1.7	0	0	0	0	19	5.9
CSF	3	2	0	0(0)	0	0	0	0	3	0.9
Sputum	3	2	10	16.9	1	1.2	2	5.6	16	5
Total	150	100	59	100	77	100	36	100	322	100

As represented in Table 4, pus isolates had the highest ESBL prevalence 55.1% among other clinical samples including urine (53.3%), blood (47.3%), CSF (33%) and sputum (25%).

**Table 4:** Distribution of ESBLs by type of specimen

	Al-Nasser		Al-Rantisi		Al-Durrah		Beit Hanoun		Total	
	N	%	N	%	N	%	N	%	N	%
Urine	63	56.2	24	57.1	33	45.8	16	56.2	136	53.3
Pus	9	64.3	3	50	2	50	2	40	16	55.1
Blood	8	44.4	1	100	0	0	0	0	9	47.3
CSF	1	33.3	0	0	0	0	0	0	1	33
Sputum	0	0	3	30	0	0	1	50	4	25
Total	81		31		35		19		166	

### Antibiotic profile

Meropenem and amikacin were the highest rates of susceptibility antibiotics against ESBLs producer as they were respectively effective in 83.1% and 82.5% of ESBL isolates. The least effective antibiotics were amoxicillin (3.1%) and cephalexin (13.9%). ESBL producing microorganisms on the other hand, showed high resistance against cefotaxime (79.5%), ceftriaxone (78.9%) and ceftazidime (79.5%). In comparison, in non-ESBL producers, meropenem and amikacin also showed high rate of sensitivity (83.3% and 80.7% respectively), while the highest rate of resistance was obtained with amoxicillin and co-trimoxazole (87.8% and 57.6 respectively; Table 5).

**Table 5:** Comparison of antibiotic susceptibility between ESBL and non-ESBL producing isolates

Antibiotic	ESBL						Non ESBL						P value
	S		I		R		S		I		R		
	%	N	%	N	%	N	%	N	%	N	%	N	
Amikacin	82.5	137	0.6	1	16.9	28	80.7	126	5.8	9	13.5	21	0.02
Cephalexin	13.8	23	0	0	86.1	143	53.2	83	0	0	46.7	73	0
Cefotaxime	18.6	31	1.8	3	79.5	132	69.8	109	4.4	7	25.6	40	0
Ceftriaxone	19.2	32	1.8	3	78.9	131	71.1	111	6.4	10	22.4	35	0
Ceftazidime	19.2	32	1.2	2	79.5	132	73.07	114	11.5	18	15.3	24	0
Cefuroxime	16.2	27	3.01	5	80.7	134	64.7	101	8.3	13	26.9	42	0
Co-trimoxazole	19.2	32	1.2	2	79.5	132	37.1	58	5.1	8	57.6	90	0
Meropenem	83.1	138	6.02	10	10.8	18	83.3	130	7.05	11	9.6	15	0.88
Ciprofloxacin	52.4	87	9.03	15	38.5	64	76.2	119	7.6	12	16.02	25	0
Doxycycline	24.6	41	6.6	11	68.6	114	42.9	67	9.6	15	47.4	74	0.001
Gentamicin	56.02	93	5.4	9	38.5	64	66.02	103	5.1	8	28.8	45	0.16
Imipenem	72.8	121	12.6	21	14.4	24	78.8	123	12.8	20	8.3	13	0.03
Amoxicillin/clauvanic acid	28.3	47	18.07	30	53.6	89	44.8	70	10.8	17	44.2	69	0.006
Amoxicillin	3.01	5	0	0	96.9	161	10.2	16	1.9	3	87.8	137	0.006

The prevalence of resistance to four or more antibiotics was 91% in ESBL producers (151/166), and 48.7% in non-ESBL producers (76/156). Furthermore, the prevalence of resistance to eight or more antibiotics was 75.3% in ESBL producers (125/166), and 17.9% in non-ESBL producers (28/156). Finally, the prevalence of MDR in ESBL isolates was 92.1% (153/166), compared to 60.8% (95/156) in non-ESBL producers.

### Genotypic characterization of ESBLs

Out of the 322 isolates, 144 were screened for CTX-M, TEM and SHV production. Using PCR, 85 isolates (59.02%) were positive for at least one of the three genes. These isolates were also positive for ESBL production using phenotyping methods.

Among the 85 isolates positive by PCR, 60% were CTX-M positive, 57.6% were TEM positive and 38.8% were SHV positive. The rate of detection of one gene, two genes or three genes was 57.6%, 28.2% and 14.1% respectively (Table 6).

**Table 6:** Prevalence of blaCTX-M, blaTEM and blaSHV genes among ESBL isolates

	N	%
blaCTX-M	515	60
blaTEM	49	57.6
blaSHV	33	38.8
1 gene	49	57.6
2 genes	24	28.2
3 genes	12	14.11
blaCTX-M + blaSHV	7	8.23
blaCTX-M + blaTEM	15	17.6
blaTEM + blaSHV	2	2.4

Table 7 shows the distribution of PCR positive isolates by hospital. CTX-M was the most prevalent ESBL gene in Beit Hanoun and Al-Rantisi hospitals, while TEM was the predominant gene in Al-Durra hospital. The same percentage was obtained for CTX-M and TEM in Al-Rantisi hospital.

**Table 7:** Distribution of ESBL genes by hospitals

	Al-Nasser		Al-Rantisi		Al-Durrah		Beit Hanoun	
	N	%	N	%	N	%	N	%
Number	95		20		17		12	
ESBL	56	58.9	10	50	10	58.8	9	75
CTX-M	34	60.7	7	70	4	40	6	66.6
TEM	33	58.9	7	70	5	50	4	44.4
SHV	23	41.1	2	20	3	30	5	55.5

At least one gene was detected in 71.2% of *E. coli* isolates, 65.5% of *Klebsiella* isolates, 10.5% of *Pseudomonas* isolates, 41.6% of *Acinetobacter* spp. isolates, 75% of *Proteus mirabilis* isolates, 66% of *Enterobacter* spp. isolates, 33.3% of *Citrobacter* spp. isolates and the only one *Serratia* isolate. In *E. coli*, TEM was the most prevalent ESBL gene (41.1%) followed by CTX-M (39.7%) and SHV (21.9%), while in *K. pneumonia* isolates the prevalence of CTX-M, TEM and SHV was (55.1%, 41.3%, and 41.3% respectively). On the other hand, in *P. aeruginosa* only SHV gene was detected (10.5%). In *Acinetobacter* spp. isolates the prevalence of CTX-M, TEM and SHV was (33.3%, 16.6%, and 8% respectively). The only one isolate of *Serratia* had only TEM gene (Table 8).

**Table 8:** Distribution of ESBL genes among ESBL microorganisms

	CTX-M		TEM		SHV		ESBL		Total
	N	%	N	%	N	%	N	%	N
*E. coli*	29	39.7	30	41.09	16	21.9	52	71.2	73
*K. pneumoniae*	16	55.1	12	41.3	12	41.3	19	65.5	29
*P. aeruginosa*	0	0	0	0	2	10.5	2	10.5	19
*S. marcescens*	0	0	1	100	0	0	1	100	1
*Acinetobacter* spp.	4	33.3	2	16.6	1	8	5	41.6	12
*P. mirabilis*	1	25	1	25	1	25	3	75	4
*Enterobacter* spp.	1	33.3	2	66.6	1	33.3	2	66.6	3
*Citrobacter* spp.	0	0	1	33.3	0	0	1	33.3	3
Total	51		49		33		85		144

## Discussion

Gram-negative bacilli that are multidrug resistant have been increasingly responsible for life-threatening illnesses all over the world [30]. Patients with ESBL-producing organisms had a considerably greater mortality rate than those with non-ESBL isolates [13].

Few studies have addressed ESBL prevalence in Gaza strip. The published information in 2008 indicates that prevalence of ESBL among 200 bacterial isolates was 22%. The rate of ESBL production among *E. coli* and *Klebsiella* spp. was (9% and 35%) respectively [31]. Another recent study showed that ESBL production among 40 clinical isolates from burn unit in Al Shifa hospital in Gaza was 37.5% [32].

Studies from the West Bank hospitals reported that the prevalence of ESBL producers among *E. coli* clinical isolates were 32.7% and 47.7% [33, 34]. El Aila reported that ESBL production was found in 45 (27%) and 11 (26.8%) of 159 *E. coli* and 41 *K. pneumoniae* isolates from Al Shifa hospital, respectively [35]. Tayeh et al., found significant percentages of ESBL-producing *K. pneumoniae* (59.3%) and *E. coli* (39.1%) among clinical isolates in another recent investigation [36]. High prevalence of ESBL was reported in Asian countries which varied from 66.7% in India [37], 54.7–61% in Turkey [38, 39], 41% in United Arab Emirates [40], and 72.1% in Iran [41].

In our study, we characterized the prevalence of ESBL among Gram negative bacilli in four pediatric hospitals in Gaza strip. To the best of our knowledge, this is the first study which address the prevalence of ESBLs among children in different pediatric hospitals in Gaza strip. In addition, ESBL genes were characterized among ESBL positive isolates.

### Phenotypic detection

Using phenotyping methods, the prevalence of ESBL producing Gram negative bacilli isolates was 51.6% (166/322). Our results were higher in comparison with Omani Children (14.9%) [42], and Chandramohan et al. who determined the prevalence of ESBLs among Enterobacteriaceae in a Texas Children’s Hospital to be (13.9%) [43].

In the present study, there was variation in ESBL production according to the type of sample and isolated microorganism. Our study showed the highest rate of ESBL production was among *K. pneumoniae* (63.4%) followed by *E. coli* (55.3%) which was very high in comparison to USA in 2004 (1.4% for *E. coli* and 4.4% for *K. pneumoniae*) and Europe (10.8% for *E. coli* and 13.6% for *K. pneumoniae*) [44]. However, our result is relatively close to that obtained by Ejaz et al., where they assessed the frequency of ESBL producing *E. coli* and *K. pneumoniae* (57.4% and 71.7%) respectively in urine samples at the Children’s Hospital and Institute of Child Health Lahore, Pakistan [45]. ESBL production among *E. coli* and *Klebsiella* spp. was high in comparison with Al Muharrmi et al. in Oman (13.3%, 16.6%) respectively [42]. ESBL producing *K. pneumoniae* were 54.4% in a study from Latin America [46]. In another study conducted in Pakistan, 56.9% isolates of *E. coli* were ESBL positive [47].

In this study, the prevalence of ESBL production was highest in pus compared to other types of specimens. Contrary to our results, Al Muharrmi reported that urine (70.8%) was the main source of ESBLs from all patients, followed by blood (15%) [42]. Degnan et al. described the proportion of children with ESBL-producing urinary isolates in Maryland, a total of 7.8% (29/370) of patients grew Gram-negative urinary isolates with an ESBL strain [48]. In a study from India, nearly 40% urinary isolates of *E. coli* and *K. pneumoniae* were ESBL positive [49]. Mekki et al. reported in Khartoum, ESBL production in 53% of *E. coli* and *and Klebsiella* species isolates from the patients suffering from urinary tract infections [30]. Dotis et al. conducted a study in Thessaloniki, Greece, and found, 48 out of 463 positive urine cultures (10.4%) were phenotypically ESBL-producing bacteria [16]. Moreover, Rezai et al. reported that of 327 uropathogen *E. coli* isolates, (30.5%) were ESBL producers [50].

Other studies reported lower rates of ESBL-producing *E. coli* in countries like India (27%), Lebanon (13.3%), Korea (9.2%), and Turkey (17%) [51, 52].

### Antibiotic profile

In our study, meropenem and amikacin were the highest rates of susceptibility antibiotics against ESBL producers (83.1%, 82.5%) respectively. While, the least effective antibiotics were amoxicillin and cephalexin (resistance rate was 96.9% and 86.8% respectively).

Cefotaxime, ceftriaxone and ceftazidime showed high rate of resistance against ESBL producing isolates (79.5%, 78.9% and 79.5%) respectively. The rate of resistance against gentamicin and ciprofloxacin was 38.5%. Chandramohan et al. showed that 100% and 98.5% of the isolates were susceptible to meropenem and amikacin, respectively, whereas only 62% were susceptible to gentamicin. In addition, 6% and 5% of the isolates were susceptible to ceftazidime and cefotaxime, respectively [43].

Another study conducted by Degnan et al. who reported that most ESBL organisms were susceptible to the tested carbapenem antibiotics of Ertapenem (100%) and Meropenem (93.8%), and most were susceptible to Amikacin (92.3%). Moreover, fewer than half of the ESBL-producing organisms tested were susceptible to gentamicin (25.8%), but more than half were susceptible to fluoroquinolones, including ciprofloxacin (68.8%) [48]. Al Muharrmi et al. detected that the carbapenems (imipenem and meropenem) were the most active antibiotics against the tested ESBLs with no resistance recorded followed by amikacin with 8% resistance. All the ESBLs were resistant to oximino-cephalosporins (cefotaxime and ceftazidime 100% resistant), gentamicin and ciprofloxacin (46%, 54% respectively) and Co-trimoxazole 62% [42].

ESBL producing *E. coli* showed the highest sensitivity for meropenem and amikacin 87.2%, while lower to cefotaxime, ceftriaxone and ceftazidime were (73.4%, 74.5%, 72.3%) respectively. The highest resistance was to amoxicillin 97.6%, and cephalexin 92.8%.

In our study ESBL producing *Klebsiella* spp. showed the highest sensitivity to meropenem and amikacin (81%, 78.6%) respectively, and higher resistance than *E. coli* to cefotaxime, ceftriaxone and ceftazidime which was (90.5%, 88.09%, and 95.2%) respectively. The highest resistance was to amoxicillin 95.7% and cephalexin 80.9%.

Ejaz et al. reported that ESBL producing *E. coli* showed maximum resistance to cefotaxime (100%), ceftazidime (99.4%) and cefuroxime (93.3%), while minimum resistance was seen with meropenem (1.3%), piperacillin/tazobactam (10.3%) and nitrofurantoin (27.6%). ESBL producing *K. pneumoniae* showed high resistance to ceftazidime (100%), cefotaxime (98.7%) and cefuroxime (98.1%) while low resistance was seen with meropenem (3.6%), piperacillin/tazobactam (17.6%), and nitrofurantoin (28.5%) [45].

In our study, gentamicin had 55.3% activity against *E. coli* compared to Europe and USA where the *E. coli* susceptibility to gentamicin was 66.7% and 80% respectively in 2004 [45].

In a study conducted in Saudi Arabia, ESBL producing *E. coli* and *K. pneumoniae*, the authors found that ESBL producing *K. pneumoniae* were resistant to meropenem (5.6%), gentamicin and piperacillin/tazobactam (11.1%) and amikacin, ciprofloxacin (6.7%) [53].

Moreover, another study by Rezai et al. who discussed ESBL producing *E. coli* in urine sample found that the ESBL isolates showed highest susceptibility to carbapenems (66%) and amikacin (58%). The highest rate of resistance was observed for the following antibiotics: cefixime (99%), colistin (82%), and ciprofloxacin (76%) [43].

### Genotypic detection

Out of the 322 isolates, 144 were screened for CTX-M, TEM and SHV production., 85(59%) have at least one gene. The 85 isolates were characterized as ESBL producer using synergy test. This result was very high in comparison with Chandramohan et al. who reported a prevalence rate of 7% (94/1430). In his study, CTX-M-type ESBLs were the most common ESBLs among the pediatric isolates and was detected in 70 of the 94 ESBL-producing isolates (74%). Only 26 isolates (27%) carried blaTEM, and only 23 isolates (24%) carried blaSHV [43].

The prevalence rate of CTX-M, TEM and SHV genes in this study was (60%, 57.6%, and 38.3%) respectively. Our results are comparable with Al Tayeb et al. who reported the prevalence rate among these genes were (75%, 61% and 38%) respectively [54].

Rezai et al., found TEM gene was the most prevalent (49%) followed by SHV (44%), CTX-M (28%), VEB (8%), and GES (0%) genes [50].

Dirar et al. reported prevalent ESBL genotypes were blaTEM (86%), blaCTX-M (78%) and blaSHV (28%). These genes were found mainly in *Escherichia coli* (38%, 37%, 2%) and *K. pneumonia* (34%, 31%, 26.1%) respectively [55]. In our study, the rate of TEM, CTX-M and SHV among *E. coli* isolates was (41.1%, 39.7%, and 21.9%) respectively.

### Limitations

It was interesting to include other genes in the study like Klebsiella carabepenem resistance genes and the subtypes of ESBL genes but due to limitation in funding resources, these tests have not been done. Also due to the absence of advanced technology in our Lab. like whole genome sequencing, we could no proceed for further characterization of antibiotic resistance genes. The study was based on phenotypic characterization of ESBL bacterial isolates in addition to PCR confirmatory test.

## Conclusion

Our results show that the prevalence rate of ESBL is increasing in the children in pediatric Gaza strip hospitals with prevalence rate 51.6%. A substantial level of resistance to first- and second-generation cephalosporin was also observed. This poses a significant problem for hospitalized children’s treatment. Furthermore, the high rate of meropenem resistance among ESBL-producing bacteria is concerning, as this antibiotic is the first-line treatment for MDR bacteria. Our findings shed more insight on the problem of antibiotic resistance among the children in Gaza strip hospitals and would be helpful for formulation of an antibiotic policy and its rational use. blaCTX-M genes are the most dominant genes in ESBLs-producing isolates. This work adds to the evidence of blaCTX-M and blaTEM’s global spread and stresses the importance of epidemiological surveillance.

## Data Availability

All data generated or analysed during this study are included in the tables provided with the manuscript.
